# Brain Stimulation Therapy for Central Post-Stroke Pain from a Perspective of Interhemispheric Neural Network Remodeling

**DOI:** 10.3389/fnhum.2016.00166

**Published:** 2016-04-21

**Authors:** Takashi Morishita, Tooru Inoue

**Affiliations:** Department of Neurosurgery, Faculty of Medicine, Fukuoka UniversityFukuoka, Japan

**Keywords:** transcranial direct current stimulation, post-stroke central pain, interhemispheric inhibition, motor cortex, pain suppression

## Abstract

Central post-stroke pain (CPSP) is a debilitating, severe disorder affecting patient quality of life. Since CPSP is refractory to medication, various treatment modalities have been tried with marginal results. Following the first report of epidural motor cortex (M1) stimulation (MCS) for CPSP, many researchers have investigated the mechanisms of electrical stimulation of the M1. CPSP is currently considered to be a maladapted network reorganization problem following stroke, and recent studies have revealed that the activities of the impaired hemisphere after stroke may be inhibited by the contralesional hemisphere. Even though this interhemispheric inhibition (IHI) theory was originally proposed to explain the motor recovery process in stroke patients, we considered that IHI may also contribute to the CPSP mechanism. Based on the IHI theory and the fact that electrical stimulation of the M1 suppresses CPSP, we hypothesized that the inhibitory signals from the contralesional hemisphere may suppress the activities of the M1 in the ipsilesional hemisphere, and therefore pain suppression mechanisms may be malfunctioning in CPSP patients. In this context, transcranial direct current stimulation (tDCS) was considered to be a reasonable procedure to address the interhemispheric imbalance, as the bilateral M1 can be simultaneously stimulated using an anode (excitatory) and cathode (inhibitory). In this article, we review the potential mechanisms and propose a new model of CPSP. We also report two cases where CPSP was addressed with tDCS, discuss the potential roles of tDCS in the treatment of CPSP, and make recommendations for future studies.

## Introduction

Stroke is a vascular disorder of the brain causing various symptoms including motor weakness, sensory disturbances, balance problems, and spasticity. Pain after stroke can be caused by various conditions secondary to spasticity, and a recent study reported that as many as 39.0% of stroke patients experienced new-onset chronic pain after stroke (Klit et al., 2011). Among various pain etiologies, central post-stroke pain (CPSP) is an especially debilitating, severe disorder characterized by intractable pain with abnormal sensations such as burning and allodynia, which severely affect the quality of life (QOL).

CPSP was first described by Dejerine and Roussy as a consequence of stroke-related lesions in the thalamus (Dejerine and Roussy, 1906); however, lesions in other brain structures in the somatosensory pathway may result in CPSP (MacGowan et al., 1997; Klit et al., 2009). In the somatosensory pathway, lesions in the ventrocaudalis portae nucleus of the thalamus and lateral medulla particularly predispose patients to CPSP (Sprenger et al., 2012). The prevalence of CPSP has been reported to be 1–12% (Andersen et al., 1995; MacGowan et al., 1997; Lampl et al., 2002; Weimar et al., 2002; Widar et al., 2002; Appelros, 2006; Kuptniratsaikul et al., 2009; Lundström et al., 2009).

Even though the mechanisms of CPSP remain unclear, CPSP has been considered to be a maladapted network reorganization problem after stroke (Hosomi et al., 2015), as CPSP usually occurs in a delayed fashion from weeks to months after the initial insult (Nasreddine and Saver, 1997). To explain the abnormal network conditions of CPSP, various circuit models have been proposed (Klit et al., 2009; Hosomi et al., 2015). In this article, we review the potential mechanisms and propose a new model of CPSP. We also report two cases where CPSP was ameliorated with transcranial direct current stimulation (tDCS) and discuss the potential roles of tDCS in the treatment of CPSP and future studies.

## Malfunctioning Neuronal Circuits

CPSP is characterized by either spontaneous or evoked unpleasant feelings described as allodynia, hyperalgesia, and dysesthesia. Insults to the central nervous system (CNS) induce various responses including neurochemical reactions, cytotoxicity, and inflammation at the cellular levels, and these changes have been considered to induce maladapted neuroplasticity resulting in the abnormal sensations of CPSP (Yezierski, 2005; Costigan et al., 2009).

Hyperactivities in pain-related structures have been described in various studies and are supported by the fact that medications suppressing neuronal activities were reported to be effective for CPSP (Leijon and Boivie, 1989; Attal et al., 2000; Vestergaard et al., 2001; Canavero and Bonicalzi, 2004; Vranken et al., 2008). In particular, spontaneous pain has been considered to be due to hyperexcitability in the pain circuits of the brain (Vestergaard et al., 1995), and neurophysiological studies revealed hyperactive thalamic bursting activities in CPSP cases (Lenz et al., 1994, 2004). These findings were also supported by neuroimaging studies that showed increased regional cerebral blood flow (rCBF) in the thalamus of patients with Wallengerg syndrome and CPSP (Peyron et al., 1998).

The mechanisms of abnormal hyperactivities in the pain network could be also explained by “disinhibition theory” (Craig and Bushnell, 1994). The CNS is controlled by a delicate balance between excitation and inhibition (Vanegas and Schaible, 2004; Hull and Scanziani, 2007; Bee and Dickenson, 2008; Costigan et al., 2009; Heinricher et al., 2009), and the pain sensations in CPSP are considered to be caused by an imbalance. Burning pain could be explained by the damage to the transmission system for cold sensations, for instance.

Conversely, it has been reported that additional stroke lesions may either aggravate (Kim, 1999) or alleviate the preexisting pain (Soria and Fine, 1991; Helmchen et al., 2002). These cases illustrated that CPSP is a network reorganization disorder. It should be noted that there are affective and sensory components in pain sensation (Sewards and Sewards, 2002a,b). Limbic structures including the amygdala and insular cortex are a part of the affective pain circuit (Price, 2000), and there is a possibility that CPSP involves the malfunctioning of the circuit. Additionally, a recent resting-state functional magnetic resonance imaging (fMRI) study has shown changes in the default mode network activities in chronic pain states (Baliki et al., 2014).

## Invasive Brain Stimulation Procedures

In a classic clinical experience, the applications of thalamotomy (Menon, 2014) and postcentral gyrectomy were described (Erickson et al., 1952). These procedures were performed based on a theory that the thalamus and somatosensory cortex are the “center of the pain perception,” and removing these structures might decrease pain sensations. These procedures are no longer performed in modern neurosurgery practice. Currently, there are two neurosurgical approaches to CPSP: deep brain stimulation (DBS) and invasive motor cortex (M1) stimulation (MCS). These brain stimulation therapies have been widely used, as they are considered to be safer than destruction surgery, due to the possibility of reversibility.

Various brain structures have been stimulated with DBS methods to treat intractable pain. The most frequently reported DBS targets have been the periaqueductal gray matter (PAG), periventricular gray matter (PVG), and ventroposterior (VP) nucleus of the thalamus (Hosobuchi, 1983; Tsubokawa et al., 1984; Owen et al., 2006). The mechanism of action of PAG/PVG stimulation was originally reported to involve activation of the μ-opioid system (Hosobuchi et al., 1977) even though increases in endogenous opioid levels were not consistently found in these cases (Dionne et al., 1984; Young and Chambi, 1987). Electrical stimulation of the VP nucleus has also been considered to suppress the abnormal firing in the thalamus. However, no randomized controlled studies have definitively demonstrated favorable outcomes with these methods (Bittar et al., 2005). In both procedures, PAG/PVG and Vc DBS leads were unilaterally implanted in the ipsilesional hemisphere. Another classic DBS target was the septal nuclei, which were considered to be associated with pleasurable feelings (Heath, 1963). However, Gol reported that electrical stimulation of the septal nuclei was only effective in one of six cases (Gol, 1967).

Recently, neuropsychiatric DBS approaches have been applied to address the affective components of pain in pain disorders. The DBS targets included limbic structures, the anterior cingulate cortex (ACC; Boccard et al., 2014a,b), and the ventral capsule/ventral striatum (VC/VS; Machado et al., 2013; Morishita et al., 2015a). The ACC stimulation was applied based on the experience of anterior cingulotomy for intractable pain and obsessive-compulsive disorder (OCD; Brotis et al., 2009). Boccard et al. reported favorable outcomes in the pain levels and QOL of 11 patients who had follow-up evaluations after bilateral ACC DBS (Boccard et al., 2014a). However, Morishita et al. (2015a) reported an unsuccessful case of unilateral VC/VS stimulation. Currently, a bilateral VC/VS DBS study is underway (clinicalTrial.gov Identifier: NCT01072656).

In the early 1990’s, MCS was first introduced by Tsubokawa et al. (1991a,b). Since then, many researchers have replicated the effects of electrical stimulation of the M1 using either invasive or non-invasive methods (Lima and Fregni, 2008). Nguyen et al. (2011) reported that MCS showed greater than 40% pain reduction on the visual analog scale (VAS) in 60% of CPSP patients in their literature review. The efficacy of MCS has been proven by several controlled trials as well (Nguyen et al., 2008; Velasco et al., 2008).

Tsubokawa proposed the descending pain inhibitory mechanism in his report and suggested that electrical stimulation of the upper level structures in the sensory pathway may inhibit deafferentation pain from lower level lesions (Tsubokawa et al., 1993). Peyron et al. (2000, 2007) revealed that MCS activated remote areas, including the cingulate gyrus. A recent animal study showed that MCS suppressed activity in the primary somatosensory cortex and prefrontal cortex (Jiang et al., 2014). Interestingly, pain relief usually is delayed several days to weeks following the start of MCS therapy (Nguyen et al., 2011). These findings may indicate that pain relief by MCS can be achieved by global pain network modulation involving corticocortical and thalamocortical loops rather than merely activating the primary M1. Katayama et al. (1998) reported that MCS more effectively addressed CPSP in patients with better motor functions. This finding may indicate that the degree of damage in the corticospinal tract (CST) is associated with the integrity of the pain inhibitory network involving the M1.

## Interhemispheric Interactions

Various animal and neuroimaging studies have shown post-stroke neuroplastic changes in the neural network involving the contralesional hemisphere (Xerri et al., 2014). For example, a recent animal study demonstrated enhanced activity in the somatosensory cortex of the contralesional hemisphere only 30–50 min after a small ischemic lesion was induced in the somatosensory cortex (Mohajerani et al., 2011). Additionally, compensatory remodeling with functional recovery reportedly occurred in the contralesional hemisphere 1 month after the functional loss of the ipsilesional hemisphere in the recovery process after complete infarction of the somatosensory cortex (Takatsuru et al., 2009).

**Figure 1:**
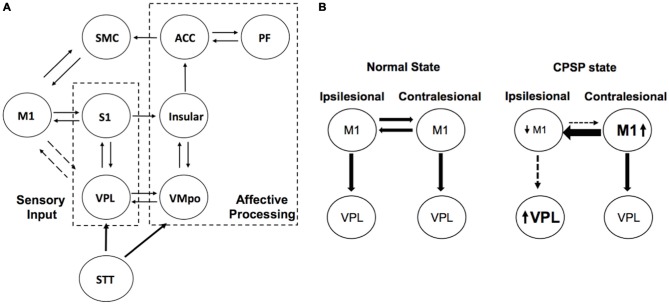
**Schema explaining interhemispheric inhibition (IHI) in central post-stroke pain (CPSP). (A)** Simplified pain circuit model composed of lateral and medial thalamic pain pathways. The motor cortex (M1)-VPL connection is described as dotted lines as there is an indirect connection. It should be also noted that there is an indirect somatosensory projection from the S1 to insular cortex through posterior parietal cortex (Price, 2000). **(B)** Impaired descending inhibition pathways from primary M1. Ipsilesional M1 activity is decreased due to not only stroke lesion but also inhibitory signals from the contralateral M1. ACC, anterior cingulate cortex; PF, prefrontal; SMC, supplementary motor cortex; STT, spinothalamic tract; VMPo, posterior ventromedial nucleus of the thalamus; VPL, ventral posterolateral nucleus of the thalamus.

fMRI studies have shown contralesional M1 activation during tasks using the impaired upper extremity in stroke cases (Rehme et al., 2011; Grefkes and Fink, 2014). Recent studies using transcranial magnetic stimulation (TMS) and MRI revealed that abnormal activity of the contralesional M1 might inhibit motor recovery after stroke (Grefkes and Fink, 2014; Volz et al., 2015), and a resting-state fMRI study revealed increased interhemispheric M1-M1 functional connectivity in stroke patients compared with that in healthy volunteers (Liu et al., 2015). All of these findings underpin the importance of the role of the contralesional hemisphere in the network reorganization after stroke. In this context, there is a possibility that the maladapted neuroplasticity in the contralesional hemisphere may partly contribute to the abnormal pain sensations in CPSP. In fact, it has been reported that additional stroke lesions in the contralateral hemisphere to the first stroke lesion influenced the preexisting CPSP (Kim, 1999; Helmchen et al., 2002). We hypothesized that the inhibitory signals from the contralesional hemisphere may suppress the activities of the M1 in the lesioned hemisphere, and therefore pain suppression mechanisms may be malfunctioning in the CPSP patients (Figure 1).

## tDCS for CPSP

As mentioned above, past studies have shown that recovery of the impaired limb may be inhibited by abnormal contralesional M1 activities. This interhemispheric inhibition (IHI) theory has been applied for neurorehabilitation therapy using tDCS to improve motor functions (Lüdemann-Podubecká et al., 2014). In tDCS therapy, the bilateral motor cortices can be stimulated simultaneously using an anode (excitatory) and cathode (inhibitory). tDCS, therefore, has been considered to be a reasonable treatment modality to address interhemispheric imbalance due to stroke. Based on the IHI theory and the fact that anodal M1 stimulation suppresses the CPSP, we considered that tDCS may address both interhemispheric imbalance in neural activities and pain at the same time.

Only a few reports have concerned the use of tDCS for CPSP, even though tDCS have widely used for the treatment of other types of neuropathic pain (Fregni et al., 2006; DosSantos et al., 2012; Mehta et al., 2015). Most studies placed the anode over the contralateral M1 to the painful site and the cathode over the supraorbital area on the other side, and continuous stimulation was administered for 20 min at 2000 μA. Bae et al. used the same tDCS method for CPSP cases and reported the clinical effects of active tDCS therapy group compared to a sham stimulation group (Bae et al., 2014). In the same report, the authors concluded that pain reduction was achieved only in the active stimulation group. Another report, from our group, showed that tDCS improved CPSP as well as motor functions, and an imaging study demonstrated improved interhemispheric balance (Morishita et al., 2015b).

Here we present two representative CPSP cases where pain reduction was successfully achieved with tDCS therapy using a commercially available stimulator (DC-Stimulator plus, neuroConn, Germany). For the tDCS procedure, we positioned the electrode aiming at the M1, and the anode and cathode were placed on the lesional and the contralesional sides, respectively, on C3 and C4 of the international 10–20 electroencephalography system. We administered 2500 μA of continuous stimulation for 20 or 25 min. These parameters were selected based on the previous tDCS report concerning safety (Poreisz et al., 2007).

**Figure 2:**
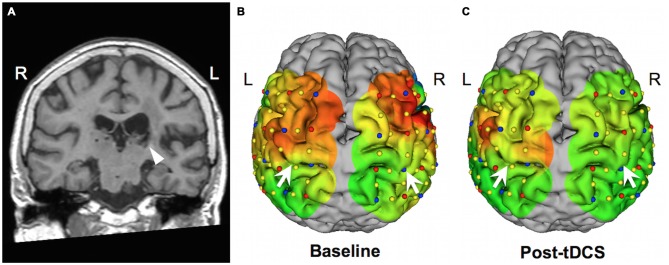
**Neuroimaging studies in case 1. (A)** Coronal view of a T1-weighted image. The arrow indicates a post-hemorrhagic lesion in the left thalamus. **(B,C)** Functional near infrared spectroscopy (fNIRS) results showing oxyhemoglobin level mapping during a right fist closure and opening task over a 3-D reconstructed image of the patient’s brain. Red and green indicate higher and lower functional activity levels, respectively. Arrows indicate the central sulci. Following all transcranial direct current stimulation (tDCS) sessions, activity in the right hemisphere was reduced. (This figure was adapted from Morishita et al. (2015b) with permission).

The first case was a 72-year-old woman with dysesthesia in her right hemibody, who had had a left thalamic hemorrhage 1 year prior. The pain started 3 months after the left thalamic hemorrhage, and she rated the pain as 60/100 on the VAS. In this case, we administered 10 sham stimulations and 10 active stimulations during 2 weeks at a hospitalized setting. Her pain level was evaluated in a double-blinded fashion, such that the rater and the patient did not know whether sham or active stimulation had been administered at each session. The pain level was significantly lower with active stimulation than sham stimulation (active vs. sham: 26.9 ± 5.49 vs. 39.5 ± 13.4, *p* = 0.006). Motor function was evaluated using an action research arm test (ARAT), which demonstrated improvement from 30 (baseline) to 37 (after all sessions). We also performed functional near infrared spectroscopy (fNIRS) to evaluate the interhemispheric balance at baseline and after all tDCS sessions. The fNIRS study showed improvement in the imbalance of the motor activity between the left and right hemispheres, and the activated motor area was more focused on the left hemisphere (Figure 2). This fNIRS finding was consistent with the results of previous fMRI studies (Grefkes and Fink, 2014). This case was previously reported elsewhere (Morishita et al., 2015b).

**Figure 3:**
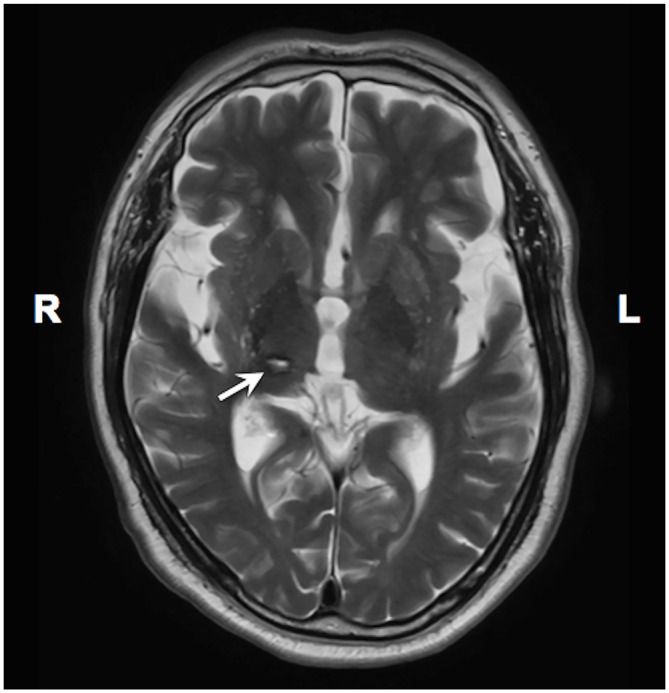
**A T2 weighted MRI image showing thalamic lesion in case 2.** An arrow indicates the stroke lesion in the right thalamus.

The second case was a 66-year-old man who started having burning pain and allodynia in his left hemibody 3 months after a right thalamic hemorrhage (Figure 3). He visited us 16 months after the onset of CPSP. We administered tDCS therapy twice a week on an outpatient basis. The tDCS settings were the same as in case 1. Before the tDCS therapy, he rated his pain in his upper extremity as a 96 on the VAS; however, he rated his pain as 48 on the VAS following 15 sessions of tDCS therapy. In this case, we evaluated the motor function of the impaired upper extremity using the Fugl–Meyer Assessment scale, and the upper extremity score improved from 57 (baseline) to 62 (after all sessions).

As presented in our illustrative cases, tDCS may be a promising treatment option for CPSP cases. Interestingly, our cases showed improvements in motor function as well as pain. It may be debated whether the motor recovery was secondary to the pain reduction or not, however, we consider that electrical stimulation of the M1 itself results in motor recovery, as shown by various studies (Lüdemann-Podubecká et al., 2014). To test our theory and prove the effectiveness of tDCS for CPSP, further clinical studies are warranted. Additionally, even though case reports are not enough convincible to conclude that addition of contralateral cathodal tDCS had any additional effect over ipsilateral anodal stimulation alone, we believe this bilateral tDCS approach may address the abnormalities in the interhemispheric neural network.

## Conclusions

In this article, we briefly reviewed the basic theories concerning the mechanisms of CPSP and proposed a CPSP neurocircuit model involving the contralesional M1. Malfunctioning neuronal circuits in CPSP may involve the contralesional hemisphere, and IHI may play an important role in pain mechanisms. Most brain stimulation therapies in the past have targeted the ipsilesional hemisphere, but we hypothesize that intervening in both hemispheres may be more effective to address CPSP. Further investigation of network abnormalities in the contralesional hemisphere may shed light on the potential mechanisms of CPSP.

Rather than trying to address the “abnormal region” in the brain, a neural network modulation approach to the global pain system would be desirable in future studies (Thompson et al., 2012). In this context, non-invasive brain stimulation techniques such as TMS and tDCS are excellent treatment options as well as research tools. Since a number of studies have already shown the efficacy of electrical stimulation of the M1 in the ipsilesional hemisphere, neuroplastic changes following magnetic or electrical stimulation of the contralesional may also be observed. Based on these findings, more effective brain stimulation parameters may be found.

Due to the heterogeneous nature of stroke, CPSP etiology varies among patients, and the number of patients who receive brain stimulation therapy is limited. Therefore, cross-over study designs having active and sham stimulation periods for each case might be desirable to test the efficacy of new stimulation approaches. For future clinical trials using brain stimulation techniques, we also propose formation of a registry database recording clinically important variables including: (1) anatomical location of the stroke lesion; (2) time between the stroke onset and CPSP onset; (3) detailed pain assessment using universal measures; (4) details of stimulation methods and parameters; and (5) clinical outcomes, inclusive of post-procedure pain scores and adverse events. This will allow us to analyze the data from a standardized cohort and lead to better understanding of CPSP etiology.

## Author Contributions

TM contributed to conception of the article and data collections, and wrote the manuscript. TI supervised the manuscript writing and reviewed the manuscript.

## Conflict of Interest Statement

The authors declare that the research was conducted in the absence of any commercial or financial relationships that could be construed as a potential conflict of interest. TM has no disclosures related to this study. TM has received grant supports from Japan Society for Promotion of Science, St. Luke Life Science Institute, Nakatomi foundation, Takeda Science Foundation, and the Uehara Memorial Foundation. He has received honoraria from Boston Scientific and Medtronic as a consultant within the past 12 months. TI has no disclosures related to this study. TI has received a grant support by the Clinical Research Promotion Foundation, Japan.
